# Comparison of ozonated water and acidified copper sulphate in prevention of digital dermatitis in dairy cows

**DOI:** 10.1186/s13028-022-00657-8

**Published:** 2022-12-20

**Authors:** Hertta Pirkkalainen, Dörte Döpfer, Timo Soveri, Minna Kujala-Wirth

**Affiliations:** 1 Department of Production Animals, Faculty of Veterinary Medicine, University of Helsinki, Paroninkuja 20, 04920 Saarentaus, Finland; 2 Department of Medical Sciences, School of Veterinary Medicine, University of Wisconsin–Madison, Madison, 53706 USA

**Keywords:** Copper sulphate, Dairy cow, Digital dermatitis, Hoof bath, Ozonated water

## Abstract

Digital dermatitis (DD) is the most significant infectious hoof disorder of cattle in Europe. Hoof baths are one of the most common control methods. Copper sulphate and formalin are commonly used in hoof baths, but their use is problematic in many European countries for health, environmental and safety reasons. Ozonated water and acidified copper sulphate were tested as prevention of DD in a 5-month study. Data were derived from 302 hind feet of Holstein and Estonian Red cows (no. of cows = 151) from a commercial dairy farm in Estonia. Altogether 168 hind feet were included in the acidified copper sulphate group and 134 feet in the ozonated water group. Hoof bathing was carried out three days a week (Mon, Wed, Fri) for two months and then two days a week (Mon, Wed) for three and a half months, in both groups. Ozonated water was sprayed on to the digital skin of hind feet of cows twice a day on treatment days, while the cows were eating. The copper sulphate bath consisted of copper sulphate (2%) mixed with an organic acid compound to acidify and ionize the solution. Cows walked through acidified copper sulphate solution twice a day on treatment days as they were exiting the milking parlor. DD negative and DD positive test results in both groups were compared and statistically tested for differences. The copper sulphate solution was more effective than ozonated water at preventing acute DD lesions. A random maximum likelihood model demonstrated that the odds ratio for DD in the ozonated water group was six times higher compared with DD in the acidified copper sulphate group. Most of the cows that were initially without any DD lesions (M0 + no other severe hoof lesion), remained lesion-free in both groups (copper sulphate group 97% and ozonated water group 88%). Despite trial design deficiencies, the findings indicate that acidified copper sulphate was a more effective solution in preventing DD than ozonated water.

## Findings

Digital dermatitis (DD) is the most significant infectious hoof disorder of cattle in Europe and North America with herd prevalence up to 97% [1, 2]. DD represents a considerable animal welfare and economic problem [3, 4]. Hoof bathing is currently one of the most common methods used to control DD. Copper sulphate (CuSO_4_) and formalin have been demonstrated to have positive effects in controlling DD [5–8] but both compounds are banned in most European countries because of their toxicity [9, 10]. Several other products at various concentrations and frequencies are also used.

During the last decade, dentistry has recognized and discussed the antibacterial effect of ozone and ozonated water. Ozone can eliminate caries pathogens, enhance epithelial wound healing and improve decontamination of the root canal [11, 12]. In vitro studies using ozonated water have shown a positive treatment effect in periodontal patients, especially against oral bacteria such as *Fusobacterium nucleatum* and *Porphyromonas gingivalis* [13]. Kontturi et al. found *Fusobacterium necrophorum*, *Porphyromonas levii* and *Treponema* species from interdigital phlegmon lesions in Finnish dairy cattle [14]. These bacteria are similar at the genus level to those found from human oral cavities.

The aim of this trial was to investigate if ozonated water is as effective compound as acidified CuSO_4_ in prevention of DD lesions.

The study was conducted on a commercial dairy farm in Estonia, which additionally provided research and teaching facilities for the Estonian University of Life Sciences. Cows (Holstein and Estonian Red) were housed indoors in a freestall barn with two separate units [milking parlor (MP); automated milking system (AMS)] that both had approximately 60 cows. Young livestock and dry cows were housed in a separate, but similar building. Cows were fed total mixed ratio *ad libitum* with additional concentrate feeders. Cubicles had rubber mattresses with peat bedding, floors were solid concrete floors covered with rubber mats and scraper moved continuously across floors. MP cows were milked twice a day in a side-by-side parlor with eight places.

Farm personnel reported all lame cows to veterinarians (n = 4), who examined them promptly in a trimming chute. A commercial hoof bath solution (4Hooves, DeLaval, Tumba, Sweden) was used regularly to control DD. MP cows entered the hoof bath when departing the milking parlor and AMS unit had a hoof bath after the milking robot. Hoof trimming was done twice a year.

At the beginning (7.11.2016) and at the end of the project (26.4.2017) all cows and heifers were inspected in a trimming chute. Feet were washed and lesions recorded and treated. DD lesions were classified into M1–M4.1 [15, 16] of which M1, M2 and M4.1 lesions were considered acute, and M3 and M4 lesions chronic [17]. Animals with acute DD between hoof trimmings, were labelled as acute DD despite of the second hoof trimming result. If a cow was moved to the dry cow group or culled, it was examined before leaving the farm. Breed, parity, parturition date, number of lactations, days in milk, culling date, reason for culling, and trial entry/exit date of cows was recorded.

A special bath with flowing ozonated water was planned to be used in AMS group. As it proved to be problematic, ozonated water was applied directly to digital skin of hind feet twice a day with a backpack sprayer (Stihl SG 71, Andreas Stihl AG & Co. KG, Waiblingen, Germany) while cows were eating. The solution was produced at the farm by Lotus pro-machine (Aktiivivesi Suomi, Mathildedahl, Finland). Oxidation-reduction potential (ORP) was measured by personnel with ORP-tester (Adwa AD14, Adwa Instruments Inc., Szeged, Hungary) every time after generating the solution. A level of > 900mV was accepted for spraying [18].

The hoof bath in the MP group consisted of 4 kg of CuSO_4_ (2%) mixed with 4 dL of organic acid (DigiDerm™, Swetrade Pharmaceuticals AB, Södertälje, Sweden) and 196 L of water. Personnel tested pH with litmus indication paper and it was maintained at pH 3.5–5.5 as recommended by Sullivan and Döpfer [19]. A fresh solution was made on every hoof bathing day.

Preventive treatment was carried out three days a week (Mon, Wed, Fri) for two months and then two days a week (Mon, Wed) for three and a half months. DD lesions were classified into five different stages [20, 21]. Acute DD included both M2 and M4.1 lesions. As suggested in the Lameness in Ruminants DD-workshop held in Japan in 2019, a good hoof bath reduces the formation of new M2 lesions, reduces transition from M4 to M4.1 and reduces transition from M4.1 into M2 [19]. In addition, proliferative M4 lesions should change into hyperkeratotic M4 lesions. As we used also local treatment for active lesions, we focused on the formation of lesions on healthy feet.

STATA IC version 16 was used for data analysis (Stata Corporation, Tx, US). Descriptive statistics and bivariate analysis were carried out to see how different factors correlated to DD lesions. Number of cows having healthy feet and different DD lesions was collected at the beginning and end of trial. These counts were then entered into a 2 × 2 contingency table. Results from the second trimming were imported into the logit model of STATA, with cow as a random factor and breed, parity, days in trial and treatment groups as predictors. Interactions were tested between predictors.

Data was collected from 340 cows. Invalid data, for example cows that had left the trial early, was not included. In total, 33 heifers were assigned to MP and 17 heifers to AMS unit. As only hind feet were treated with ozonated water, data was derived from 302 hind feet (cow, n = 151). See Table 1.

**Table 1:** Digital dermatitis lesion results from hind feet at the beginning and at the end of trial for acidified CuSO_4_ and ozonated water groups

Lesion	Number of lesions in acidified CuSO_4_ group in the beginning of trial	Number of lesions in acidified CuSO_4_ at the end of trial	Number of lesions in ozonated water group in the beginning of trial	Number of lesions in ozonated water group at the end of trial
Healthy (M0) (%)	145 (86.3%)	140 (88.3%)	117 (87.3%)	104 (77.6%)
Acute DD M2^a^ and M4.1^a^ lesions (%)	12 (7.4%)	7 (4.2%)	15 (11.2%)	20 (14.9%)
M3^a^ lesions, hypertrophic M4^a^ lesions (%)	8 (4.8%)	15 (8.9%)	1 (0.8%)	7 (5.2%)
Proliferative M4^a^ lesion (%)	3 (1.8%)	6 (3.6%)	1 (0.8%)	3 (2.2%)

Digital dermatitis (DD) lesion results from hind feet at the beginning and at the end of trial for acidified CuSO_4_ and ozonated water groups. No M1 lesions were found. If a cow had an acute DD lesion between examinations, it was calculated among the lesions at the end of trial

^a^Based on reports from Döpfer et al. [20], modified by Berry et al. [21]

Breed and parity had no statistically significant effect in our random model nor showed up in the descriptive analysis. The factor ‘days in trial’ was similar for most of the cows. Results were looked based on DD results but also after removing other severe lesions (no SU, WLD or tyloma = unaffected).

Of the unaffected hind feet at the beginning of trial, 77 (74.7%) feet in CuSO_4_ group and 58 (69.9%) in ozonated water group remained without any DD lesions. Only one unaffected foot contracted acute DD in CuSO_4_ group, compared to 10 hind feet in ozonated water group. The differences between groups were statistically significant (Pearson’s chi-squared P = 0.002).

**Fig. 1:**
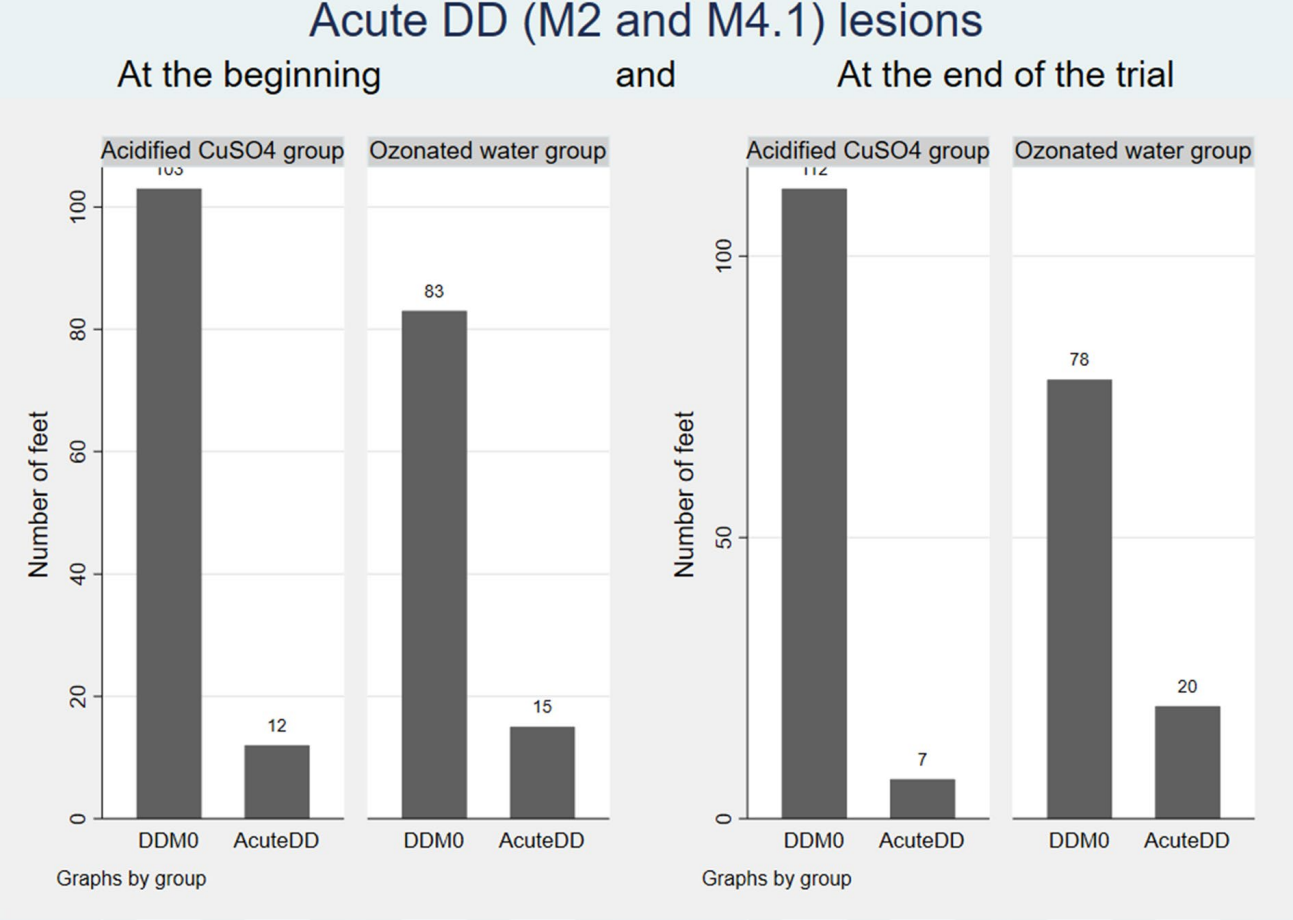
DDM0 and acute DD (M2 and M4.1) lesions at the beginning and end of trial. Number of healthy feet (no digital dermatitis, sole ulcer, white line disease or tyloma; referred as DDM0 in the figure) and feet with acute DD (M2 and M4.1) lesions at the beginning and end of trial in different treatment groups

In Fig. 1 unaffected feet and acute DD feet are presented at the beginning and at the end of the trial in both groups.

A stepwise approach was chosen to build a model for DD in this trial, for testing the model and adding cow as a random factor. The random maximum likelihood model demonstrated that test group was a significant factor, and “days in trial” was maintained as a confounder. Because of small sample size and missing values, the model fit was not optimal. Odds ratio for acute DD when exposed to ozonated water was 6.03 times higher compared with being exposed to CuSO_4_ (CI 1.18–30.91, P = 0.031). Heifer status (primiparous vs. multiparous) was also tested but being a heifer did not affect DD prevalence.

There could be several possible explanations for ozonated water being less effective than CuSO_4_. First, the two groups were exposed to the compound differently. Hoof bath length and number of steps a cow takes defines the contact time for the solution. The recommended length is 3–3.7 m [22]. In CuSO_4_ group hoof bath length was 2 m, but hooves were completely immersed in hoof bath solution, which also had a minor cleaning effect. Spraying did not cover the entire hoof and did not remove all manure. Therefore, ozonated water was probably unable to reach bacteria located deep within the skin and covered by a protective layer of manure. Cleaning with pressure might have an influence and we suggest this to be investigated further. There was one M2 lesion in the front feet in both groups at the beginning of trial. We did not consider this a major source of infection as these lesions were also treated. However, we must acknowledge that in AMS group the front feet did not receive any spraying.

Cows in the ozonated water group had more acute DD lesions at the beginning of the trial (11% vs. 7%). This might have resulted in a greater infection pressure. Many of the healthy feet remained without DD lesions in both groups. Because of low number of cows, this could be a coincidence or due to genetic predisposition [23].

Due to difficulties in adapting heifers to use AMS, we lost random selection of heifers. This should have been planned better beforehand. However, being a heifer, compared to being a multiparous cow, did not have a significant effect on the occurrence of acute DD lesions. One explanation could be a shared dry cow group, where heifers have encountered DD bacteria before moving to lactating group.

Though lame cows were examined in the trimming chute between the beginning and the end of trial, we might have missed some DD lesions as not all cows with DD are lame [24].

It is possible that ozonated water is comparable with other disinfectants and might be used when DD prevalence is not particularly high. However, the cost of the machinery was moderately high. Using ozonated water for disinfection of hoof trimming chutes or hoof trimming equipment might be worth investigating. The use of a 2% CuSO_4_ with 0.2% organic acid was a novel combination which proved to be effective. In other studies, the amount of organic acid has been much higher [6, 8], but the pH of hoof bath solutions has not been highlighted. Using hoof baths with low pH (< 2) has been associated with higher acute and proliferative DD incidence than a higher pH (> 3) [19]. By measuring pH and using reasonable amount of acid, hoof baths can be made safer and more effective to cows.

The findings indicate that on farms with high acute DD prevalence acidified CuSO_4_ is a better preventive treatment option than ozonated water. However, ozonated water spraying represents an alternative preventive measure.

## Data Availability

The data that support the findings of this study are available from the corresponding author upon reasonable request.

## References

[1] Holzhauer M, Hardenberg C, Bartels CJ, Frankena K (2006). Herd- and cow-level prevalence of digital dermatitis in the Netherlands and associated risk factors. J Dairy Sci.

[2] Jacobs C, Orsel K, Barkema HW (2017). Prevalence of digital dermatitis in young stock in Alberta, Canada, using pen walks. J Dairy Sci.

[3] Losinger WC (2006). Economic impacts of reduced milk production associated with papillomatous digital dermatitis in dairy cows in the USA. J Dairy Res.

[4] Cha E, Hertl JA, Bar D, Gröhn YT (2010). The cost of different types of lameness in dairy cows calculated by dynamic programming. Prev Vet Med.

[5] Logue DN, Gibert T, Parkin T, Thomson S, Taylor DJ (2012). A field evaluation of a footbathing solution for the control of digital dermatitis in cattle. Vet J.

[6] Laven RA, Hunt H (2002). Evaluation of copper sulphate, formalin and peracetic acid in footbaths for the treatment of digital dermatitis in cattle. Vet Rec.

[7] Speijers MHM, Finney GA, McBride J, Watson S, Logue DN, O’Connell NE (2012). Effectiveness of different footbathing frequencies using copper sulfate in the control of digital dermatitis in dairy cows. J Dairy Sci.

[8] Holzhauer M, Bartels CJ, Bergsten C, van Riet MMJ, Frankena K, Lam TJGM (2012). The effect of an acidified, ionized copper sulphate solution on digital dermatitis in dairy cows. Vet J.

[9] Flemming CA, Trevors JT (1989). Copper toxicity and chemistry in the environment: a review. Water Air Soil Pollut.

[10] Swenberg JA, Moeller BC, Lu K, Rager JE, Fry RC, Starr TB (2013). Formaldehyde carcinogenicity research: 30 years and counting for mode of action, epidemiology, and cancer risk assessment. Toxicol Pathol.

[11] Huth KC, Paschos E, Brand K, Hickel R (2005). Effect of ozone on non-cavitated fissure carious lesions in permanent molars. A controlled prospective clinical study. Am J Dent.

[12] Nogales CG, Ferrari PH, Kantorovich EO, Lage-Marques J (2008). Ozone therapy in medicine and dentistry. J Contemp Dent Pract.

[13] Eick S, Tigan M, Sculean A (2012). Effect of ozone on periodontopathogenic species—an in vitro study. Oral Investig.

[14] Kontturi M, Junni R, Simojoki H, Malinen E, Seuna E, Klitgaard K (2019). Bacterial species associated with interdigital phlegmon outbreaks in finnish dairy herds. BMC Vet Res.

[15] Dopfer D, Holzhauer M, Boven M (2012). The dynamics of digital dermatitis in populations of dairy cattle: model-based estimates of transition rates and implications for control. Vet J.

[16] Biemans F, Bijma P, Boots NM, de Jong MCM (2018). Digital dermatitis in dairy cattle: the contribution of different disease classes to transmission. Epidemics.

[17] Döpfer D, Vanhoudt A. M-stage decision tree. In: Proc 12th international conference of lameness in ruminants, Tokyo, Japan. 2019.

[18] Suslow TV. Oxidation-reduction potential (ORP) for water disinfection monitoring, control, and documentation. 2004. https://escholarship.org/uc/item/1730p498#author. 10.3733/ucanr.8149. Accessed 3 Oct 2021.

[19] Sullivan J, Döpfer D. The effect of hoof bath pH value on improved claw health with special focus on digital dermatitis in dairy cattle. In: Proc 12th international conference of lameness in ruminants, Tokyo, Japan. 2019;190.

[20] Döpfer D, Koopmans A, Meijer FA, Szakáll I, Schukken YH (1997). Histological and bacteriological evaluation of digital dermatitis in cattle, with special reference to spirochaetes and *Campylobacter faecalis*. Vet Rec.

[21] Berry SL, Read DH, Famula TR, Mongini A, Döpfer D (2012). Long-term observations on the dynamics of bovine digital dermatitis lesions on a California dairy after topical treatment with lincomycin HCl. Vet J.

[22] Cook NB, Rieman J, Gomez A, Burgi K (2012). Observations on the design and use of footbaths for the control of infectious hoof disease in dairy cattle. Vet J.

[23] Schöpke K, Gomez A, Dunbar KA, Swalve HH, Döpfer D (2015). Investigating the genetic background of bovine digital dermatitis using improved definitions of clinical status. J Dairy Sci.

[24] Frankena K, Somers JGCJ, Schouten WGP, van Stek J, Metz JHM, Stassen EN (2009). The effect of digital lesions and floor type on locomotion score in dutch dairy cows. Prev Vet Med.

